# Patient Power and Empowerment: Mitigating Elements of Valuable Patient Participation in Healthcare Collaboratives

**DOI:** 10.3390/bs13040347

**Published:** 2023-04-21

**Authors:** Anja Minheere, Wim Lambrechts, Jelle Mampaey, Talia Stough, Marjolein C. J. Caniëls, Janjaap Semeijn

**Affiliations:** Faculty of Management, Open Universiteit of the Netherlands, 6419 AT Heerlen, The Netherlands

**Keywords:** power inequities, empowerment, interrelation dynamics, collaboration, mutual trust, individual competence

## Abstract

During the last decade, the public healthcare sector has had to deal with increased competition, a growing influence of patient associations, and a necessity to deliver health services more efficiently and effectively. Despite recognising the patient participant’s role as a critical stakeholder in value creation, there is a limited body of research on the influence and power of patient participants. This article focuses on regional health improvement collaboratives that aim to develop coordinated, multi-stakeholder solutions to their healthcare cost and quality problems. They meet regularly and include health professionals, health insurance providers, and patient participants. In this article, we explore the relationships between these stakeholders and patient participants’ interpersonal dimensions regarding empowerment and valuable collaboration. Data were collected through stakeholder observations during meetings of three regional health improvement collaboratives, as well as through semi-structured interviews with the patient participants involved in these cases. Results show that patient participants can be empowered on a personal level. However, this does not imply that patient participants are empowered within the group dynamics. Interpersonal relationships constitute a crucial hidden aspect of building trust. More dialogue and inquiry are needed to examine how patient engagement is enacted and positioned within healthcare collaboratives.

## 1. Introduction

Healthcare costs are rising, and customers are demanding more personalised as well as better care [1]. Market-like regulation has been introduced, with the expectation to stimulate competition between healthcare providers to reduce costs [2]. Healthcare organisations adopted network or supply chain (management) practices to manage these new conditions, referred to as healthcare supply chains. In healthcare supply chains, the patient should be seen as the customer. Consequently, patients are increasingly involved in healthcare innovation projects, as they are primary stakeholders.

Studies describe the importance of patient empowerment. However, there is a lack of knowledge about ‘how’ this empowerment can be achieved [3,4,5,6]. Few studies have investigated the relationships between, and among, the core co-creation values, co-creation behaviours, and supportive behaviours [3]. Patient empowerment in health literacy, patient participation, shared decision making, treatment control, and communication with health professionals may positively affect healthcare delivery [4]. The World Health Organization (WHO) also confirms the importance of patient empowerment and has set patient empowerment as the primary goal of the WHO’s 2020 Health Program [5].

This study analyses patient participation in regional health improvement collaboratives (RHIC), focusing on the power/empowerment and the interrelation dynamics of patient participation. RHICs have three defining characteristics: first, an RHIC is a non-profit organisation in a specific geographic region of the country. Second, it is governed by a multi-stakeholder board composed of healthcare providers, health insurance providers or governments, and health consumers. Third, an RHIC helps the stakeholders identify opportunities to improve health and healthcare within the community and facilitates the planning and implementation of strategies for addressing those opportunities. An RHIC provides a neutral, trusted mechanism through which all key healthcare stakeholders in the community—physicians, hospitals, health plans, employers, and patients—develop coordinated, multi-stakeholder solutions to their healthcare cost and quality problems [7]. In the Netherlands, the concept of RHIC is considered essential to successful healthcare reform and has been widely adopted. Unfortunately, there is no central registration for RHICs in the Netherlands. As limited research has addressed how patient empowerment can be achieved in healthcare, research about the functioning of RHICs is especially suitable to fill this gap. By showing how patient participants can exert influence and power in these collaboratives, we garnish insights into how valuable collaboration can be established to avoid ineffective patient involvement.

The remainder of the article is as follows: Section 2 presents a literature review. Then, the methodology of this research is outlined in Section 3. After sharing the results of observations of three RHICs and interviews with three patient participants in Section 4, a discussion follows in Section 5. Finally, conclusions and recommendations are described.

## 2. Literature Review

### 2.1. Stakeholder Involvement in Healthcare Systems

In the past, customers, i.e., patients, were considered passive recipients of complex services [8]. Today, the healthcare system can be conceived as a cluster of public and private services with the involvement of various stakeholders [9]. Furthermore, healthcare providers experienced a shift from the goods-centred to the service-centred view, focusing on identifying and developing core competencies for achieving competitive advantage by developing relationships with key economic actors in the supply chain (e.g., customers and suppliers) [10]. Due to these changes, healthcare organisations realise the importance of a customer-oriented business approach [11]. The World Health Organization [12] points out that ‘community participation’ is widely thought to improve healthcare delivery and health equity. Furthermore, healthcare practice and academia have recognised and embraced customers’ active role in co-creating the healthcare service experience [11,13].

Patient participation can be interpreted as a form of stakeholder involvement. Stakeholder theory draws on four social sciences—sociology, economics, politics, and ethics—to inform how organisations interact with stakeholders [14]. In the context of healthcare, a stakeholder is “an individual or group who is responsible for or affected by health- and healthcare-related decisions that can be informed by research evidence” [15] (p. 986).

### 2.2. Collaboration and Value Co-Creation

In healthcare, the concept of co-production, co-creation, and collaboration (although more often phrased in terms of patient participation, patient engagement, or patient and public involvement) is receiving increasing attention. Co-production of value replaces the “old” ideas of professional dominance and paternalism, conceiving the patient as a co-creator of value rather than a consumer of health services [16,17]. However, the co-creation of value depends on information symmetry, mutual trust, and equity.

Legnick-Hall [17] identified information asymmetry as one of the most significant barriers in the value co-creation process. Information sharing is essential to stakeholders for two reasons. Information sharing reduces uncertainty and enables stakeholders to understand and control their co-creation environments. Furthermore, information sharing allows stakeholders to master their role as value co-creators. If stakeholders fail to provide accurate information, the quality of value co-creation may be low [18].

Mutual trust is also a necessary condition in a successful value co-creation process. Trust increases when one party has confidence in a supply chain partner’s reliability and integrity [19]. It is an iterative process because the development of trust causes a feeling of safety and loyalty among stakeholders [3]. Engdahl and Lidskog [20] state that trust develops through emotional involvement and sensemaking. Therefore, trust is the currency that turns co-creation relationships into success [9].

Equity is the third critical condition to value co-creation. Adams [21] posited, in his equity theory, that an individual feels treated fairly when his efforts are equivalent to those of other stakeholders. Equity is based on subjective perceptions rather than absolute value and can be experienced differently by stakeholders. Ross and Kapitan [22], concluded that stakeholders desire an equitable distribution of benefits and costs to stay motivated. Therefore, if stakeholders sense inequity in engagement, it will not be easy to gain power. To summarise, we identify information symmetry, equity, and trust as vital elements to a valuable collaboration.

### 2.3. Power and Empowerment

Power is a central element in supply chain relationships. A frequent concern in management is how to gain and use power with other parties in the supply chain [23]. Djellouli et al. [24] found that if the involved public felt they did not influence the decision-making process, they sought alternative routes to voice their views. Power must also be studied in the complex interrelationships in health collaboratives, and value distribution in a single relationship might thus fall short of capturing the complexity [25]. Furthermore, interdependence fosters a commitment to meaningful collaboration. It is possible to build trust in situations of high interdependence. By contrast, where interdependence is weak, it is challenging to develop trust [26].

Garnishing insights into the mediating role of individual empowerment in patient participation is the crux of this research. Psychological empowerment is a term introduced by Zimmermann [27] in their nomological network of empowerment at the individual level. Psychological empowerment consists of three interconnected components. First, the individual feels personal control in his/her approach to matters important to him/her (interpersonal component). Second, an empowered person has a greater critical awareness to see and oversee vital issues/within and within his environment (interactional component). Third, an empowered person addresses topics necessary to him/her (behavioural component) [28]. In recent scientific articles, psychological empowerment has been renamed personal or individual empowerment. Patient empowerment refers to patients’ control over their health and ability to be more involved in their healthcare. Empowerment enables patients to manage their healthcare and advocate for themselves as the user of healthcare services [6].

**Figure 1:**
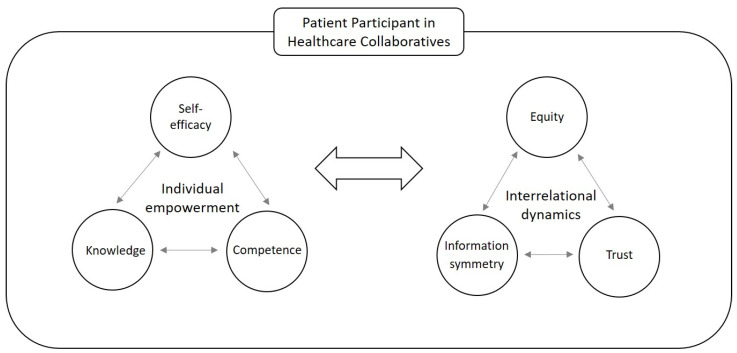
Conceptual model for the process of empowerment (source: own work, based on [30]).

In their seminal contribution, Cattaneo and Chapman [29] illustrate the key components of the process of empowerment. The authors posit that the interaction between self-efficacy, knowledge, and competence is the crux of empowerment as a process. Self-efficacy (an individual’s beliefs about his or her abilities) lies at the core element of the individual empowerment process. Locke and Latham [30] state that self-efficacy is a moderator between goals and performance. Self-efficacy represents a positive belief and was defined in the organisational context by Stajkovic and Luthans [31] as “an individual’s convictions (or confidence) about his or her abilities to mobilize the motivation, cognitive resources, and courses of action needed to successfully execute a specific task within a given context” [31] (p. 65). Knowledge is defined as understanding the relevant social context, including the power dynamics at play, the possible routes to goal attainment, the resources needed, and ways to obtain them [29]. Knowledge of power systems improves empowerment processes, such as intrapersonal, interactional, and behavioural empowerment [32,33,34]. Competence is achieved when a proper interpretation of information leads to understanding and knowledge of the issues [35]. Cattaneo and Chapman [29] articulated competence separately from knowledge, given that conceptually knowing what to do is not the same as knowing how to use this knowledge. The Empowerment Process Model [29] informs our research design, as well as the collection of our data (see Figure 1).

However, the distribution of power influences relationship dynamics, and ultimately, the empowerment of participants. Specifically, power inequities are likely to affect trust development, a vital aspect of relationships [36]. In healthcare supply chains, structures can exist that perpetuate existing stakeholder inequalities [30]. Therefore, stakeholders who lack power before participating might gain much power as a result of engagement. This dependence asymmetry of power inequalities may contribute to damaging collaboration in the stakeholder relationship [24]. Fumagalli et al. [6] suggested that delegating responsibilities to patients creates an illusion of power and is instead a reinforcement of top-down control mechanisms. If, however, a stakeholder is individually empowered, the interaction with other stakeholders and the group changes and might contribute to offsetting the issue of dependence asymmetry.

### 2.4. Conceptual Model

Although RHICs are seen as a possible mechanism for developing coordinated, multi-stakeholder collaboration to co-create value (e.g., reduce costs, improve quality, etc.), the literature indicates that achieving valuable collaborations in a context of power inequities is complex. This tension is what drives our research aim: to better understand the empowerment (or lack thereof) of patient participants in healthcare-related collaboratives and how this is related to the ability of the collaborative to co-create value.

Understanding the interaction of interrelational dynamic elements (information symmetry, mutual trust, and equity) with individual empowerment (self-efficacy, knowledge, and competence) in collaborative contexts lies at the heart of this research (as illustrated in Figure 1). In this study, we will explore these elements and their interactions in the context of patient participation in regional healthcare collaborations (RHICs) in the Netherlands.

## 3. Methods

This study aims to assess how patient participation in RHICs is practiced critically. It is executed by using an exploratory multiple case study design. Case studies help clarify an issue, problem, or phenomenon [37]. In this case study, the analysis units are stakeholders acting in an RHIC and the patient participants involved.

### 3.1. Research Design

The southern Netherlands is challenged with affordability and accessibility of care due to an aging population, unemployment, and poor health outcomes. It is not a coincidence that RHICs established in this region are trying to deal with these challenges. In order to select and reach out to RHICs, we collaborated with Burgerkracht Limburg (Citizenpower Limburg), an independent network organisation dedicated to the interests of all (vulnerable) citizens in the province of Limburg. As the specific focus of our study is on power/empowerment and the interrelation dynamics of patient participation, RHICs were selected that have existed for more than two years, are fully functional, and are committed to the Triple Aim Initiative. The Triple Aim Initiative strives to: improve patients’ care experience (including quality, access, and reliability); improve populations’ health; and reduce the per capita cost of healthcare (or at least lower the rate of increase). At least one financer, one health professional, a project manager, and a patient participant should be involved in each RHIC. RHICs with other compositions, new RHICs, or RHICs not committed to the triple aim initiative were excluded from this study. Five RHICs met all of these inclusion and exclusion criteria, all of which were approached for our study. One RHIC was discontinued before the study commences, and one did not give permission to conduct observations or interviews. The remaining three RHICs gave permission for observations and interviews.

The RHIC represented in Case 1 is a mental health foundation that meets once a year to make procurement decisions for regional care. The RHIC represented in Case 2 aims to improve health in a small region in the North of Limburg. At the time of the observations, they were working on qualitative pharmaceutical care at a lower cost and started a project to substitute more expensive statins (which help lower cholesterol in the blood) with generic medicine. Case 3 is an RHIC funded as a pioneering project. Stakeholder representatives run pioneer sites. These pioneer sites seek the best organisational and management structures to work with and explore new forms of funding (such as shared savings). Case 3 is the longest-running collaborative in the sample (i.e., in a mature phase). Case 1 is an established collaborative but not as long-running as Case 3 (i.e., in a consolidation phase). Case 2 is the newest collaborative in the sample (i.e., still in the initiation phase). Table 1 presents the composition and members of the three RHICs in our study.

### 3.2. Data Collection

Data were collected by performing observations of meetings and conducting semi-structured interviews. Participants were asked for consent to be observed, interviewed, or both (video/audio recording of each). The written consent was stored. The observations occurred after desk research and contact with the project managers (telephone or online meetings). We conducted our observations during regular, scheduled meetings of the involved RHICs. These meetings typically lasted between 65 and 100 min and were filmed, followed by detailed verbatim transcription of spoken words and non-verbal signs. Field notes were also taken. The filmed observations were also studied without sound. Subsequently, the patient participants attending the meeting were interviewed. Each patient participant is an experienced expert, between 35 and 65, who participates voluntarily, without payment. The interviews were conducted in person and lasted between 45 and 60 min. The interviews were semi-structured, following a guide based on the literature review. 

Participant observation is a specific approach to gaining knowledge based on direct contact between the researcher and the social objects of interest. In this kind of observation, it is important to be unbiased and focus on directly observable events [38]. Therefore, a guideline for observation objectives was used to observe the group dynamics.

### 3.3. Data Analysis

Operationalised constructs for the observations and interviews were derived from the literature and geared towards exploring the relationships between stakeholders in RHICs, as well as patient participants’ interpersonal dimensions regarding empowerment and collaboration. As discussed above, information symmetry, mutual trust, and equity are crucial to gain power and co-create value in collaborative contexts. Given the power differences among actors in healthcare organisations, mechanisms should be in place to minimise inequity [39]. These mechanisms aim to give equal opportunities for each stakeholder to participate at various stages of the decision-making process. Furthermore, each stakeholder has a clearly defined and enshrined role in priority-setting rules and guidelines. Finally, there should be ensured accessibility of relevant information to each stakeholder to reduce information asymmetries and an ongoing engagement of stakeholders to build trust over time [38]. Wallerstein [40] found that effective empowerment depends on the agency and leadership of the people involved in the overall context in which they occur. The literature also shows that leaders play a crucial role in encouraging prosocial, collaborative behaviours that contribute to the growth and strengthening of networks [41]. Therefore, the components of leadership and relationship are included as observation objectives. Likewise, transparency is essential to establish information symmetry, but it also plays a role in trust building. Su, Fang, and Young [42] empirically emphasised transparency’s critical role in enhancing supply chain partnerships. Transparency is therefore added as an observation objective. 

Self-efficacy, knowledge, and competence are vital elements of individual empowerment. Locke and Latham [30] identified self-efficacy as a moderator between goals and performance and specify motivation as an offshoot of highly valued goals for which a person has high self-efficacy. Cattaneo and Chapman [29] defined knowledge as an understanding of the relevant social context, including the power dynamics at play, the possible routes to goal attainment, the resources needed, and ways to obtain them. The authors articulated competence separately from knowledge because it is conceptually distinct—knowing what to do is not the same as knowing how to do it.

A coding and pattern-matching process was used as an analysis technique. Coding helps to interpret the data and to connect the pieces of information [43]. Coding reports and field notes were analysed and coded using open thematic analyses. The results were discussed within the research group to reach a consensus and ensure interrater reliability. Quotes were selected to describe the perception of the patient participants in terms of motivations, influence, and suggestions for change. The thematic codes included patient participant; belief in oneself, action; critical awareness; motivation; emotional involvement; sense making; leadership; decision making; problem solving; health insurance representative; health professional; project manager; all information shared; participation; explanation; non-verbal signs; use of voice; other member behaviour; shake hands; and private talk (displayed in Table 2 and Table 3).

The pattern that emerged was compared with relevant literature. Every effort was made to ensure this study’s validity and reliability [44]. A transparent chain of evidence was provided by explaining the data collection circumstances, specific procedures, and data analysis procedures. Internal validity refers to causal relationships between variables and results [44], and we derived this by specifying a transparent research model. We seek to ensure reliability through the transparency and clarity of our research design and methods, which are enhanced through careful documentation and clarification of the research procedures.

## 4. Results

In this section, we describe evidence of individual empowerment (or lack thereof) and the group dynamics occurring in each of the three RHICs based on the observations and interviews. Table 2 displays the frequency of elements observed during meetings. Table 3 displays the frequency of elements garnished through interviews. Based on the patterns that emerged in the data, the three cases included in our study constitute three types of patient participation in healthcare collectives. In Case 1, the patient participant was individually empowered, and the collective was in a consolidation stage. In Case 2, the patient participant was individually empowered, but the collaborative was in the initial stage of development. In Case 3, the patient participant was individually empowered, and the collective was in a mature stage of development. The three cases are described individually in Section 4.1, Section 4.2 and Section 4.3, and the collective insights are synthesised in Section 5.

### 4.1. Case 1: Individually Empowered Patient Participant in a Consolidated Collaborative

In Case 1, stakeholders sought information from other members, and the perceived self-efficacy of the patient participant was high, as PP1 stated, “I am the best candidate” and “This work has given my recovery a boost because I have to look at my illness with a helicopter view”. PP1 took a class and has a colleague patient participant whom he consults if necessary: “When I have questions about procedures or rules, I often call X”. PP1 would like to see other patient participants take a more active role: “I will try to motivate other PP to participate in this group”.

At the beginning of one of the observed meetings, one of the stakeholders asked for advice about a topic irrelevant to this project, asking, “Do you know the * checklist?” (all information shared). Three other members confirmed. “Is it a useful tool? I have heard negative comments at a symposium” (participation). Another member wanted to know how family counselling is organised in the mentioned organisation. A group participant explained this with help from a third member. Thus, information symmetries and exchanges were present in this group. The insurance company members showed interest in the opinion of the patient participant: “Can you tell us how this is done in this organization? (…) Thank you very much for your explanation” (explanation). 

Regarding company-related information, the insurance company members only shared information after detailed questions and remained rather superficial when they asked, “Are you satisfied with the use of e-health in your organization?” They added, “We are extremely interested in the patient’s opinion” (all information shared). Unfortunately, no explanation followed why they are interested (all information shared).

Non-verbal signs informed us about interdependency and transparency related to mutual trust. Interdependency in this RHIC is underpinned by a need for information, which only the patient participant can deliver. Positive non-verbal signs outweighed the negative ones by far for this collective. For example, a favourable sitting position, which is active, open, and turned towards the speaker, was noticed twelve times. Opposing sitting positions such as hanging on the chair or aggressive (arms tight-folded) were found less frequently. Arms fast-folded were seen in the patient participant arguing about an important topic. After approving nods from other stakeholders and smiles, the patient participant leaned back in his chair again. In the interview, the patient participant said that he felt capable of the job, well educated, and prepared but noticed he also noted feelings of insecurity and the need to be reassured, asking, “Did I do well during the meeting?” (non-verbal signs; belief in oneself).

Trust is also noticeable, indicating that the patient participant is supported. The participant spoke fast and hesitated at the start of the meeting, and then the health professional helped him/her by summarising and checking with the patient participant whether the summarisation was correct. The other members supported him/her by active listening and positing, with faces turned towards the speaker and many confirming nods. As a result, the patient participant became more confident (observed by speaking at tempo and in a more relaxed sitting position). The patient participant said in the interview, “During the first meetings, I often had to ask for explanations, but I felt confident to do so” (belief in oneself).

The long existence of this supply chain, the open atmosphere, and the shared goal to improve health in this organisation added to the individual stakeholders’ unique knowledge and gave credence to value creation in this supply chain. Notable, however, is the remark of one of the participants of the health insurance company. She discussed her long relationship in this network with her company’s board and obtained the answer “Nice, but we do not want you to become too familiar”. This remark makes the equity in this RHIC unclear. To what extent does the health insurance company desire fairness or equity in its relationship with the other stakeholders?

### 4.2. Case 2: Individually Empowered Patient Participant in a Newly Established Collaborative

In Case 2, information symmetry was observed. All participants understood each other, and when asked for clarification, were provided an explanation. “Stakeholder… gives information about the benefits of the chosen medicine and explains the decision tree for this kind of medication” (explanation). During the lengthy meeting, medical terms needed to be explained only twice, indicating that health literacy was not an issue in this group. PP2 prepared in advance for the meeting, saying “The documents arrive on time and when I have questions, I will get them answered before the meeting”.

Nevertheless, patient participation is new in this RHIC. The patient participant describes it as a “fight for my position” (action). Interpersonal relationships differ in this group; a few stakeholders worked together on another project with opposite interests. This tension is noticed during the observations. The noted non-verbal signs confirmed this: six positive and seventy-eight negative non-verbal signs were recorded, such as looking at a mobile phone, turning red, and not looking at the speaker. During the speaking time of one of the stakeholders, another member checked his mobile phone, and the person speaking looked down. Noted are four or five key speakers who talk most of the time, plus interruptions of speakers. Compared with the other observed cases, more negative use of voice aspects were noticed in this RHIC: five negatives and two positives. One of the members emphasised four times that “It is important to make the right choices at the start of the project”.

Contrary to Cases 1 and 3, the word “you” was used frequently in this RHIC, referring to other stakeholder groups in this RHIC. For example, “It would be best if you, health professionals, did not only care about the cure; budget control is also one of your tasks”. The use of “we” and “you” indicates tension between the different stakeholder groups. Non-verbal signs observed such as turning red, crossing arms, or taking an upright sitting position confirmed this tension. In the interview, the patient participant said, “This network can improve through better communication” (critical awareness) and additionally said, “More appreciation would be welcome” (sense making). PP2 stated that “It [referring to Patient and Public Involvement] has not been done before, and everything that happens here needs patients’ involvement”. The patient participant believed in the capability to execute the tasks and believed that she was individually empowered: “They embraced my proposal, so yes, I have influence”.

Stakeholders are prepared when the meeting starts and are willing to execute tasks. They are motivated to make this RHIC successful, shown by the passionate way of arguing (loud voice, big arm gestures, and long speaking times), but there is frustration over only achieving two subprojects. Subproject three had to be rewritten during the three years due to lower priorities set by insurance companies. Two pharmacists said, “It is a pity that the original subproject three had to be changed”, and another stakeholder said, “I, also, am not happy that the original plan has been changed. This project has been tough, but that is inherent in such projects” (other member behaviour). The project leader confirmed this, saying, “Yes, it is a pity, and it is good that we have changed it to another type of medication so that we can bring measurable results” (project manager).

Interesting is the remark of the patient participant: “We, as patient participants, belong in this healthcare supply chain, and that is where we are used for, but sometimes we are also used to reduce the tension between members”. She also said “Patient participation is new in this group”, and, “In this project, patient participation started hesitantly. I had to convince the members of my added value. It is my task to seduce the other stakeholders to look with the eyes of a patient” (action). This struggle with her position is also observed; the frequency of the project manager or other stakeholders interrupting her is significantly higher than the frequency that other stakeholders were interrupted. Thus, in this RHIC, the stakeholders struggle with their role and position and the patient participant’s role and position. Furthermore, problematic interpersonal relationships play an inhibitory role in mutual trust building. Equity and mutual trust are imbalanced in this group.

### 4.3. Case 3: Individually Empowered Patient Participant in a Mature Collaborative

In Case 3, information symmetry and equity are balanced. There is a willingness to share information, e.g., “I would like to inform you about a personnel change in my department” (participation). “All three external parties need to be present at the upcoming meeting. Is party three an important one?” Another member answers, “No, with parties 1 and 2, you cover the central area.” “Do we agree to invite parties 1 and 2?” (participation). Everybody nods or says yes. Part of the meeting time is devoted to sharing an example of a not-appropriate functioning healthcare supply chain and comparing it with this project. This group scored the highest amount (180) of coded participation items. PP3 stated, “I gained understanding and knowledge about how things work in healthcare (…) If you are willing to search for information eventually you will find it”.

There is group spirit, and the patient participant feels welcome and appreciated. The patient participant noticed that “The commitment of the members and their willingness to see the need to change, despite own interests that conflict with it, makes this network successful” (motivation). Observations confirm this: before the meeting starts, all participants greet each other and exchange informal information. Case 3 is the only observed RHIC where members choose a seat randomly and do not take the seat next to their professional colleagues. PP3 confirms, saying “I have noticed that the members listen to me”.

In this group, a professional also participates on behalf of patients. She is the representative of a public organisation stakeholder in this RHIC. She asks for background information and reacts, saying that “It is a pity that only professionals are interviewed and no public or patients” (explanation) and asking “would it be wise to speak with these people individually?” (participation). Patient and professional patient participants actively participate in the meeting and highlight the perspective of the patient but are not explicitly asked to give more information about topics.

## 5. Discussion

Despite the recognised importance of healthcare supply chains to create value and the patient participants’ role as critical stakeholders in this context, there is a limited body of research on how the collaboration of stakeholders in RHICs is conducted. This study aimed to fill this gap by analysing individual power/empowerment and interrelational dynamics in RHICs. The most notable result of our study is that when comparing the interview data with the data obtained from the observations, all patient participants reported feeling empowered in interviews, but there was mixed evidence from observed group dynamics observed. Secondly, the data from the observed cases reveal that group dynamics in collaboratives might go through phases of maturity, and interpersonal dynamics can evolve as this occurs. In this section, we discuss the individual empowerment of patient participants in collaboratives, the interrelational dynamics of such collaboratives, and the influences of the maturity/context of collaboratives on these factors.

### 5.1. Individual Empowerment

Individual empowerment is vital for patient participants to collaborate and co-create. Mann et al. [45] stated that patient participants, who have experience contributing to health research projects and have a good understanding of research, appear to be more confident to comment on the research and challenge the design. They conclude that participants need a certain level of literacy to contribute. In our observations, the patient participants are personally empowered by emotional involvement, information access, and good preparation for the task. They possess critical awareness and are experienced patient participants. The level of health literacy is also at hand. They indeed act all with confidence.

**Figure 2:**
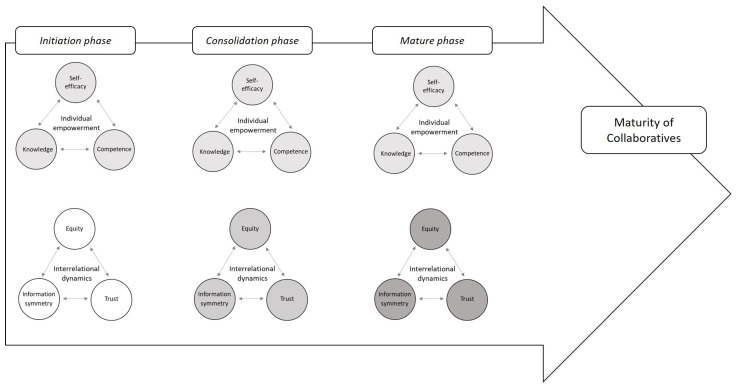
Results: individual and interrelational elements of the three RHICs in our study (darker shading denotes high levels of empowerment at the individual and group level).

Despite the self-reported empowerment of patient participants, we noticed tensions and discrepancies between the self-perceived role and individual empowerment and the observed group dynamics during the RHIC meetings. Patient participants faced the challenge of defending their status as potential change agents within the healthcare system. Ewert and Evers [9] described challenges for patient participants: they must serve the needs of patients and the public. The patient participants represent a large group of patients and the public, with little chance of becoming co-producers. Even when the patient participant performs an ideal job in both these tasks, there is the danger that they will function solely as ordinary customer services added to make modern healthcare schemes more usable but have little say in their design and implementation. Speer [34] concluded that people can feel empowered without understanding how to act on that feeling to change their communities’ conditions. Therefore, although all patient participants in this study are personally empowered, this does not automatically mean empowerment within the RHIC group dynamics. We capture this in Figure 2 by denoting that the level of individual empowerment of patient participants can be disconnected from the level of interrelational dynamics in collaboratives.

### 5.2. Interrelational Dynamics

Mutual trust and equity differed between stakeholders in the observed cases as reciprocal relationships differed. A strong trust relationship between health professionals and patient participants was found in Case 1. In Case 2, tensions were noticed between health professionals and members of the health insurance company. Additionally, trust between patient participants, health professionals, and insurance company members was also weak. In Case 3, both relationships had a solid trust base. These findings are similar to those of Groenewegen, Hansen, and De Jong [46], who found a stable and mutual trust relationship between patients and healthcare providers and weak trust between healthcare providers and insurers and between patients and insurance organisations. 

Brett et al. [47] stated that the success of patient and public involvement often relies on the interaction between individuals involved. While personal relationships between stakeholders are not explicitly included in the study design, observations showed that a tense relationship between two individual stakeholders hampered mutual trust building and true collaboration. Schruijer [48] stated, “There is a host of research done within the domain of project management, e.g., on trust, but again, hardly anything on group dynamics and how to develop collaborative relationships” (p. 18). This study confirms that personal relationships influence group dynamics and trust building, an essential topic for future research.

Trust and power are inextricably linked, and one must be considered in the presence of the other [49]. Theory suggests that power is developed and exercised through relationships [50]. Interpersonal trust facilitates and encourages communication, and repeated communication creates trust. In Case 2, mutual trust and equity are weak, and interrelational tension is observed due to a problematic collaboration of some stakeholders in another project. Jagosh et al. [51] found that “Healthy conflict, resistance, negotiation, and consensus-building are integral to establishing trust and rapport among stakeholders. Alternatively, while rare in the documented data, unresolved conflicts led to disaffection and a breakdown in the trusting relationship between stakeholders” (p. 335). Unfortunately, in Case 2, no noticeable action was undertaken to improve trust. Therefore, there is a risk of decreased trust and power indifferences.

Tokenism can occur when participants of diverse backgrounds collaborate and do not understand each other [52]. Critical conditions are building sustainable relationships and a willingness for mutual learning [53]. In Case 2, we observed weak mutual trust and a lack of conflict-solving or trust-building activities. Therefore, in Case 2, the risk of tokenism is observed. Interdependency is found in Case 1 but must be clarified in Case 2 and Case 3. If alternative venues exist where stakeholders can pursue their goals unilaterally, collaborative governance will only work when stakeholders perceive themselves as highly interdependent [26]. In Case 3, data show an active and valued patient participant. However, patient participation is more complex than simply bringing a qualified participant into the RHIC. For example, Tummers et al. [16] described that establishing a co-creating partnership between patients and healthcare professionals involves conflicting beliefs. Therefore, patient involvement can lead to struggles rather than co-production, thus provoking value co-destruction. In our study, such provocative co-destruction tensions have not been observed.

### 5.3. Maturity/Context of Collaboratives

Sufficient time is essential for collaborations to successfully mature. The RHIC in Case 2, existing for a relatively brief time (two years), seemed to struggle with collaboration. Case 3, as a pioneer site, had more time to develop relationships and participatory methods. They decided to continue the partnership after a successful period as a pioneering project. This aligns with Allen et al. [54] who emphasised that interpersonal relationships are the heart of the participatory process and that these interpersonal relationships require time to evolve. Participants need time to work on the process and become comfortable. In Figure 2, we capture this evolution in interrelational dynamics as a collaborative matures.

Another major influence on the success of collaboratives is the context in which they are operating. In Case 2, one subgoal was not achieved due to changed politics (external forces), and some stakeholders in the group felt frustrated. As a result, group cohesion was negatively impacted. Watson and Foster-Fischman [55] stated that power is context-specific and alternative settings such as collaborative decision making are particularly vulnerable to contextual shifts. Brett et al. [47] stated that contextual factors and the interaction of individuals involved cannot be underestimated in developing positive impacts. For this reason, studying context and process factors in more depth is worthwhile. 

## 6. Conclusions

In this study, we found that a patient participant needs to be individually empowered to collaborate with other stakeholders and achieve co-creation. The patient participant must be capable, representable, and qualified to accomplish this individual empowerment. To achieve this, the establishment of a patient participant profile is beneficial. Adequate funding, interaction time, and trust with patient participants can help negotiate roles, balance power, and lead to meaningful patient collaboration. We highlighted trust and equity’s importance in developing meaningful collaboration. We observed that interrelational dynamics and interpersonal relationships are vital for establishing mutual trust, and when there are unsolved issues between stakeholders, mutual trust building is complex. To avoid tokenistic patient participation in RHICs, stakeholders must be willing to build sustainable relationships, have the intention to be genuinely engaged, and be open to mutual and reciprocal learning. Furthermore, we found a discrepancy between perception and observations. Therefore, future research should thoroughly describe the context to convey more clearly what works, why it works, and who created what value.

This study provides important insight into our understanding of patient participation in RHICs. However, we acknowledge that there are limitations to this study. First, because the number of cases we observed is limited, the three cases described in this study cannot represent all RHICs, and this study needs to be replicated with a more substantial number of cases.

Second, although we observed some challenges and tensions in collaborative processes, the observed cases are relatively positive examples of co-creation. For several reasons, some RHICs did not want to participate in our study. Some of these initiatives had been put on hold or indicated that participants did not feel comfortable being observed. Researching the dark side of patient participation and collaboration and the consequences of failed co-creation would be interesting.

Third, although the community plays a role in value co-creation in regional health improvement collaboratives, we did not investigate the role of macro-level actors, such as the policy decision-makers of the regional healthcare system or the local government. Therefore, future research is needed to examine the influence of community and government policy on adopting and implementing co-creation initiatives in RHICs.

To summarise, this paper contributes to understanding the role and influence of patient participation in RHICs. Despite the uncertainties, as described in this article, and a fragile evidence base, patient engagement has become widely accepted. Considering these gaps, it would be hard to disagree that patient engagement represents a critical democratic and ethical practice in modern healthcare. Patient participation in RHICs can address the complex challenges of creating responsive and actionable healthcare evidence [56]. Successful collaboration in RHICs, with meaningful patient participation, is challenging. Therefore, more dialogue and inquiry are needed to address these gaps and precisely examine how patient engagement is enacted and positioned within healthcare supply chains in general and in RHICs.

## Figures and Tables

**Table 1:** Stakeholders in the observed RHICs.

RHIC	Total of Stakeholders Present (n)	Project Manager (n)	Health Insurance Representative (n)	Health Professional (n)	Patient Participant (n)
Case 1	6	1	2	2	2 *
Case 2	11	2	2	6	2 *
Case 3	14	1	2	9	2 **

* One participant was absent in the observed meeting. ** One of the patient representatives is a professional in patient participation.

**Table 2:** Observation objectives; numbers indicate the frequency of observed item.

Observation Objectives	Case 1 (n)	Case 2 (n)	Case 3 (n)
Knowledge sharing/collaboration			
All information shared	12	3	12
Participation	47	127	180
Health literacy			
Language/technical jargon	0	2	1
Explanation	15	18	14
Role leadership			
Project manager behaviour	7	19	25
Other members behaviour	0	16	22
Personal skills			
Time spoken	1	3	0
Use voice	6	7	5
Responsibility			
Prepared	3	1	5
Willing to execute tasks	1	4	0
Relationship			
Shakes hand	2	6	2
Makes eye contact	13	23	22
Interrupts speaker	0	7	4
Sitting position	16	11	9
Private talk/business talk	1/1	5/7	10/16
Other non-verbal signs	38	93	84

**Table 3:** Interview objectives. Numbers indicate the frequency of mentioned item by the interviewee.

Interview Objectives	Case 1	Case 2	Case 3
Self-efficacy			
Motivation	3	2	5
Believe in oneself	12	4	6
Emotional involvement	5	11	8
Sensemaking	5	5	2
Knowledge			
Critical awareness	4	7	6
Leadership	4	4	3
Action	4	3	5
Competence			
Decision making	1	1	1
Problem solving	4	5	3

## Data Availability

Due to the nature of this research, data is not shared publicly.
